# Pseudorabies Virus Inhibits Expression of Liver X Receptors to Assist Viral Infection

**DOI:** 10.3390/v14030514

**Published:** 2022-03-03

**Authors:** Yi Wang, Guo-Li Li, Yan-Li Qi, Li-Yun Li, Lu-Fang Wang, Cong-Rong Wang, Xin-Rui Niu, Tao-Xue Liu, Jiang Wang, Guo-Yu Yang, Lei Zeng, Bei-Bei Chu

**Affiliations:** 1 College of Veterinary Medicine, Henan Agricultural University, Zhengzhou 450046, China; anjxasmwy@sina.com (Y.W.); g2914026145@sina.com (G.-L.L.); lily1570304118@sina.com (Y.-L.Q.); liliyun20221186@yeah.net (L.-Y.L.); wanglufang1@aliyun.com (L.-F.W.); raewang616@outlook.com (C.-R.W.); niuxinrui2022@yeah.net (X.-R.N.); liutaoxue201516@yeah.net (T.-X.L.); wangjiang@henau.edu.cn (J.W.); 2 Key Laboratory of Animal Biochemistry and Nutrition, Ministry of Agriculture and Rural Affairs of the People’s Republic of China, Zhengzhou 450046, China; yangguoyu@henau.edu.cn; 3 Key Laboratory of Animal Growth and Development, Zhengzhou 450046, China; 4 International Joint Research Center of National Animal Immunology, Henan Agricultural University, Zhengzhou 450046, China; 5 College of Animal Science & Technology, Henan University of Animal Husbandry and Economy, Zhengzhou 450047, China

**Keywords:** pseudorabies virus, Liver X receptors, clathrin-coated pits, viral entry

## Abstract

Pseudorabies virus (PRV) is a contagious herpesvirus that causes Aujeszky’s disease and economic losses worldwide. Liver X receptors (LXRs) belong to the nuclear receptor superfamily and are critical for the control of lipid homeostasis. However, the role of LXR in PRV infection has not been fully established. In this study, we found that PRV infection downregulated the mRNA and protein levels of LXRα and LXRβ in vitro and in vivo. Furthermore, we discovered that LXR activation suppressed PRV proliferation, while LXR inhibition promoted PRV proliferation. We demonstrated that LXR activation-mediated reduction of cellular cholesterol was critical for the dynamics of PRV entry-dependent clathrin-coated pits. Replenishment of cholesterol restored the dynamics of clathrin-coated pits and PRV entry under LXR activation conditions. Interestingly, T0901317, an LXR agonist, prevented PRV infection in mice. Our results support a model that PRV modulates LXR-regulated cholesterol metabolism to facilitate viral proliferation.

## 1. Introduction

Pseudorabies (PR), also called Aujeszky’s disease, is a highly infectious disease caused by the PR virus (PRV), which is a member of the subfamily Alphaherpesvirinae of the family Herpesviridae. The genome of PRV is approximately 143 kb encoding at least 70 open reading frames [1]. PRV can infect a wide variety of mammals, including pigs, sheep, and cattle, causing severe clinical symptoms and death [2]. Although scientists have been trying to develop diagnostic approaches and vaccines in recent years, PR remains an important infectious disease that is prevalent in many countries. Several recent reports have suggested that PRV can cause human endophthalmitis and encephalitis [3,4,5]. These findings indicate that PRV infection is a potential public health risk and not limited to the swine industry. Therefore, new methods are urgently needed to prevent PRV infection.

The liver X receptors (LXRs), a family of transcription factors in the nuclear receptor superfamily, are ligand-activated transcription factors and pivotal regulators of cholesterol and lipid metabolism [6,7]. Two LXR subtypes, LXRα and LXRβ, have been identified. While LXRβ is expressed ubiquitously, LXRα is expressed highly in the liver, spleen, intestine, heart, and macrophages [8]. Ligand binding to LXR results in the formation of a heterodimer between LXR and the retinoid X receptor (RXR). The LXR/RXR complex then binds to the LXR-response elements in the promoter, initiating the transcription of target genes [9,10]. The natural ligands of LXR have been identified as oxysterols, such as 22(R)-hydroxycholesterol (22R-HC). LXR plays important roles in lipid metabolism. A number of small molecules of LXR agonists and reverse agonists, such as LXR-623, T0901317, GW3965, and SR9243, have been developed to treat lipid disorders and atherosclerosis in clinical trials [11].

Lipids are essential components for cellular and viral membranes. It has been revealed that lipids are required for the entire life cycle of a virus, including attachment, entry, genome replication, assembly, and release [12]. LXR is required for efficient replication of a number of viruses, including Newcastle disease virus (NDV) [13], human immunodeficiency virus (HIV) [14], hepatitis C virus (HCV) [15], hepatitis B virus (HBV) [16], murine gammaherpesvirus 68 [17], coxsackie B3 virus [18], and chikungunya virus [19]. However, the role of LXR in regulating PRV replication has not been documented. Here, we examined the effects of LXR on PRV replication. We demonstrated that LXR activation, which led to reduced cellular cholesterol levels, suppressed PRV proliferation. LXR agonists, such as T0901317, dramatically decreased PRV entry via interference with the cholesterol-dependent dynamics of clathrin-coated pits (CCPs). Our data suggest that LXR agonists have potential as antivirals for the control of PRV infection.

## 2. Materials and Methods

### 2.1. Mice

We purchased female 6–8-week-old BALB/c mice from the Center of Experimental Animal of Zhengzhou University (Zhengzhou, China). Mice were housed in a specific pathogen-free animal facility at Henan Agricultural University. Animal experiments were performed in accordance with protocols approved by the Use of National Research Center for Veterinary Medicine (Permit 20180521047).

### 2.2. Cells, Viruses, and Plasmids

Porcine kidney epithelial PK-15 (CCL-33, ATCC), porcine alveolar macrophages 3D4/21 (CRL-2843, ATCC), and human cervical cancer HeLa (CL-82, ATCC) cells were grown in monolayers at 37 °C under 5% CO_2_ in DMEM (Gibco, Waltham, MA, USA) supplemented with 10% FBS (Gibco), 100 U/mL penicillin, and 100 μg/mL streptomycin sulfate (Sangon, Shanghai, China). Virus titers were determined by the 50% tissue culture infective dose (TCID_50_) assay, which was calculated with the Reed–Muench method.

The virulent PRV isolate QXX (PRV-QXX) was kindly donated by Yong-Tao Li from the College of Veterinary Medicine, Henan Agricultural University [20]. The recombinant PRV strain of PRV-GFP, derived from the PRV Hubei strain with the TK gene replaced by a GFP expression cassette from the pEGFP-N1 plasmid, was kindly donated by Han-Zhong Wang from Wuhan Institute of Virology, Chinese Academy of Sciences [21]. 

Full-length porcine AP2B1 cDNA was cloned into the mCherry-N1 expression plasmid using the BamHI and KpnI restriction sites.

### 2.3. Chemicals and Antibodies

Cholesterol, T0901317, GW3965, LXR-623, and SR9243 were ordered from MedChemExpress (Monmouth Junction, NJ, USA). Filipin complex and 22-R-hydroxycholesterol were ordered from Sigma-Aldrich (St. Louis, MO, USA). Anti-LXRα, anti-LXRβ, anti-AP2B, and anti-β-actin were ordered from Proteintech (Rosemont, IL, USA); anti-ABCA1 was ordered from Novus Biologicals (Littleton, CO, USA); Alexa-Fluor-488-conjugated goat anti-mouse IgG, Alexa-Fluor-568-conjugated goat anti-mouse IgG, and Alexa-Fluor-568-conjugated goat anti-rabbit IgG were ordered from Thermo Fisher Scientific (Waltham, MA, USA). Antiserum against PRV glycoprotein gB and gE was generated by immunization of mice with purified recombinant gB and gE.

### 2.4. Cell Viability Assays

PK-15 cells were seeded at 1 × 10^4^ per well in 96-well plates. On the next day, the medium was changed to DMEM/10% FBS supplemented with various concentrations of LXR agonists for 24–48 h. CCK-8 (10 μL, DingGuo, Beijing, China) was then added to each well, and the cells were incubated for 3 h at 37 °C. Absorbance was detected at 450 nm with a microplate reader (Varioskan Flash; Thermo Fisher Scientific).

### 2.5. Flow Cytometry Assay

For green fluorescent protein (GFP) reporter assays, PK-15 cells were infected with PRV-GFP [multiplicity of infection (MOI) = 0.01] for 36 h. Cells were digested with trypsin-EDTA (Gibco), collected by centrifugation, and suspended in PBS. The percentage of GFP-positive cells was measured by flow cytometry on a Beckman CytoFLEX instrument (Brea, CA, USA). All data were analyzed with CytExpert software 2.0.

### 2.6. Immunoblotting Analysis

Cells were collected by centrifugation and lysed in RIPA buffer (Beyotime, Shanghai, China) in the presence of protease and phosphatase inhibitor cocktail (MedChemExpress). The protein concentration was determined by a BCA Protein Assay Kit (DingGuo). Equivalent amounts of total protein (30 μg) were subjected to SDS-PAGE for immunoblotting. The target proteins were detected with specific primary antibodies and appropriate horseradish peroxidase (HRP)-conjugated secondary antibodies. Visualization was performed with Luminata Crescendo Western HRP Substrate (Millipore, Billerica, MA, USA) on a GE AI600 imaging system.

### 2.7. Cell Surface Biotinylation Assay

Cells were incubated in ice-cold PBS-CM (0.1 mM CaCl_2_, 1 mM MgCl_2_) in the presence of 1 mg/mLEZ-Link NHS-biotin (Thermo Fisher Scientific) for 30 min at 4 °C. After two washes with ice-cold PBS-CM, cells were cultured in medium containing 10 mM glycine for 30 min at 37 °C. Cytosolic and membrane fractions were prepared as previously described [22]. Briefly, the cells were homogenized by passing through a #7 needle 30 times in 0.5 mL of homogenization buffer (10 mM HEPES [pH 7.4], 10 mM KCl, 1.5 mM MgCl_2_, 5 mM sodium EDTA, 5 mM sodium EGTA, 250 mM sucrose) supplemented with protease and phosphatase inhibitors (MedChemExpress) and centrifuged at 1000× *g* at 4 °C for 7 min. The pellet, containing the crude nuclear fraction, was discarded, and the supernatant was centrifuged at 12,000× *g* at 4 °C for 15 min. The resulting supernatant was the cytosol, and the pellet containing the membrane fraction was dissolved in lysis buffer (10 mM Tris-HCl [pH 6.8], 100 mM NaCl, 1% SDS, 1 mM EDTA, 1 mM EGTA) supplemented with protease and phosphatase inhibitors (MedChemExpress). Each fraction was incubated with NeutrAvidin-agarose (Thermo Fisher Scientific) and rotated for 2 h at 4 °C. After three washes with homogenization buffer (10 mM HEPES pH 7.4, 10 mM KCl, 1.5 mM MgCl_2_, 5 mM EDTA, 5 mM EGTA, 250 mM sucrose), biotinylated proteins on NeutrAvidin–agarose were eluted with SDS-PAGE sample buffer and subjected to immunoblotting analysis.

### 2.8. Quantitative Real-Time PCR (qRT-PCR)

Total RNA was extracted using TRIzol Reagent (TaKaRa, Shiga, Japan) and then reverse-transcribed with a PrimeScript RT reagent Kit (TaKaRa). qRT-PCR was performed in triplicate using SYBR Premix Ex Taq (TaKaRa). Data were normalized to the expression of the control gene encoding *β-actin*. The relative expression changes were calculated by the The 2^−ΔΔCT^ method. Quantification of the genome copy number of PRV was performed as previously described [23]. Primers used for qRT-PCR analysis were as follows: porcine *β-actin*-Fw: 5′-GCACAGAGCCTCGCCTT-3′, porcine *β-actin*-Rv: 5′-CCTTGCACATGCCGGAG-3′; porcine *Lxra*-Fw: 5′-CGTCCACTCAGAGCAAGTGT-3′, porcine *Lxra*-Rv: 5′-CAGATCTCAGAGAGCAGCGG-3′; porcine *Lxrb*-Fw: 5′-ACGCTACAACCACGAGACAG-3′, porcine *Lxrb*-Rv: 5′-CGGTGGAAGTCATCCTTGCT-3′; porcine *Abca1*-Fw: 5′-ATGGATCACTGCCCCAGTTC-3′, porcine *Abca1*-Rv: 5′-ATGTCCGCGGTGTTCTGTTT-3′; porcine *Abcg1*-Fw: 5′-GTGTACTGGATGACGTCGCA-3′, porcine *Abcg1*-Rv: 5′-CGAAGCTGACGAAGAACCCT-3′; PRV *gB*-Fw: 5′-CTCGCCATCGTCAGCAAPRV-3′, PRV *gB*-Rv: 5′-GCTGCTCCTCCATGTCCTT-3′; mouse *β-actin*-Fw: 5′-CCCCATTGAACATGGCATTG-3′, mouse *β-actin*-Rv: 5′-ACGACCAGAGGCATACAGG-3′; mouse *Lxrα*-Fw: 5′-CTGATTCTGCAACGGAGTTGT-3′, mouse *Lxrα*-Rv: 5′-GACGAAGCTCTGTCGGCTC-3′; mouse *Lxrb*-Fw: 5′-GCCTGGGAATGGTTCTCCTC-3′, mouse *Lxrb*-Rv: 5′-AGATGACCACGATGTAGGCAG-3′.

### 2.9. RNA Interference (RNAi)

Cells were transfected with the indicated siRNAs (GenePharma, Shanghai, China) using Lipofectamine RNAiMAX Reagent (Invitrogen, Waltham, MA, USA) according to the manufacturer’s instructions. The medium was replaced with DMEM containing 10% FBS at 8 h post-transfection. The knockdown efficacy was assessed by immunoblotting analysis at 48 h post-transfection. The siRNA sequences were as follows: negative control (NC): 5′-UUCUCCGAACGUGUCACGU-3′; siLXRα: 5′-CCCACGGAUGCUAAUGAAAUU-3′; siLXRβ: 5′-UCCCGCGAAUGCUGAUGAAUU-3′.

### 2.10. Fluorescence Recovery after Photobleaching (FRAP)

HeLa cells were transfected with the AP2B1-mCherry plasmid for 24 h. After 8 h of pre-treatment with the indicated compound, the cells were bleached at maximum laser intensity for 30 s in a region of 7 × 7 μm^2^ and then imaged for 5 min at 37 °C. CCP dynamics was defined by the fluorescence recovery of AP2B1-mCherry, which was performed on a Zeiss LSM 800 confocal microscope.

### 2.11. Histological Analysis

Animal tissues were fixed in 4% paraformaldehyde and embedded in paraffin. The paraffin blocks were sectioned (7 μm) for hematoxylin-eosin staining.

### 2.12. Statistical Analysis

All data were obtained from three independent experiments for quantitative analyses and expressed as the mean ± standard error. All statistical analyses were performed with a two-tailed Student’s *t* test. Significant differences relative to the corresponding controls were accepted at * *p* < 0.05. For mouse survival studies, Kaplan–Meier survival curves were generated and analyzed for statistical significance.

## 3. Results

### 3.1. PRV Infection Inhibits LXRα and LXRβ Expression In Vitro and In Vivo

**Figure 1:**
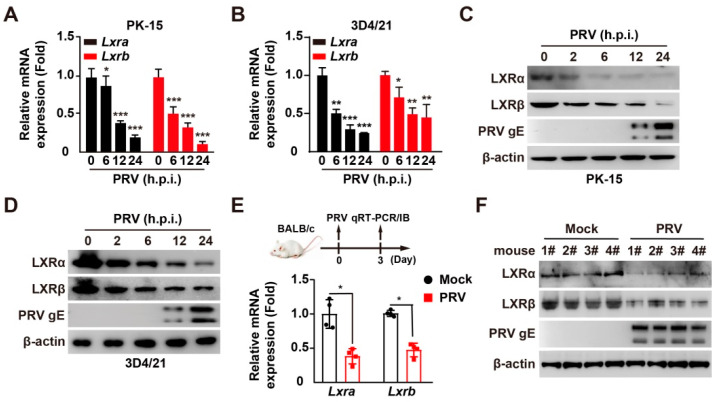
PRV infection downregulates LXR expression. (**A**,**B**) PK-15 (**A**) and 3D4/21 (**B**) cells were infected with PRV-QXX (MOI = 0.1) for 0–24 h. The mRNA levels of *Lxra* and *Lxrb* were assessed by qRT-PCR analysis. * *p* < 0.05, ** *p* < 0.01, *** *p* < 0.001. (**C**,**D**) PK-15 (**C**) and 3D4/21 (**D**) cells were infected with PRV-QXX (MOI = 0.1) for 0–24 h. LXRα, LXRβ, and PRV gE were assessed by immunoblotting analysis. (**E**) Mice were mock infected or intranasally infected with PRV-QXX (5 × 10^3^ TCID_50_/50 μL per mouse) for 3 days. The mRNA levels of *Lxra* and *Lxrb* in the lung were assessed by qRT-PCR analysis (*n* = 4 per group). * *p* < 0.05. (**F**) Mice were treated as in G. LXRα, LARβ, and PRV gE in the lung were assessed by immunoblotting analysis (*n* = 4 per group).

To determine the role of LXR in PRV infection, we evaluated the expression of LXR under PRV challenge in vitro. Cells were infected with PRV-QXX for 0–24 h, and cells were processed to measure the mRNA and protein levels of LXRα and LXRβ. PRV infection caused a reduction in *Lxra* and *Lxrb* mRNA in PK-15 and 3D421 cells (Figure 1A,B). Consistent with the mRNA levels, LXRα and LXRβ protein levels in PK-15 and 3D421 cells were all downregulated in response to PRV infection (Figure 1C,D). These results suggested that PRV inhibited LXRα and LXRβ expression in vitro.

We verified whether PRV reduced LXR expression in vivo. Mice were mock infected or intranasally infected with PRV-QXX for 3 days, and the lungs were assessed for mRNA and protein levels of LXRα and LXRβ by qRT-PCR and immunoblotting analysis. PRV infection resulted in a >two-fold decrease in *Lxra* and *Lxrb* mRNAs as compared to that in mock-infected lungs, as well as the protein levels of LXRα and LXRβ (Figure 1E,F). These data indicated that PRV infection suppressed LXR expression both in vitro and in vivo.

### 3.2. Inhibition of LXR Increases PRV Infection

**Figure 2:**
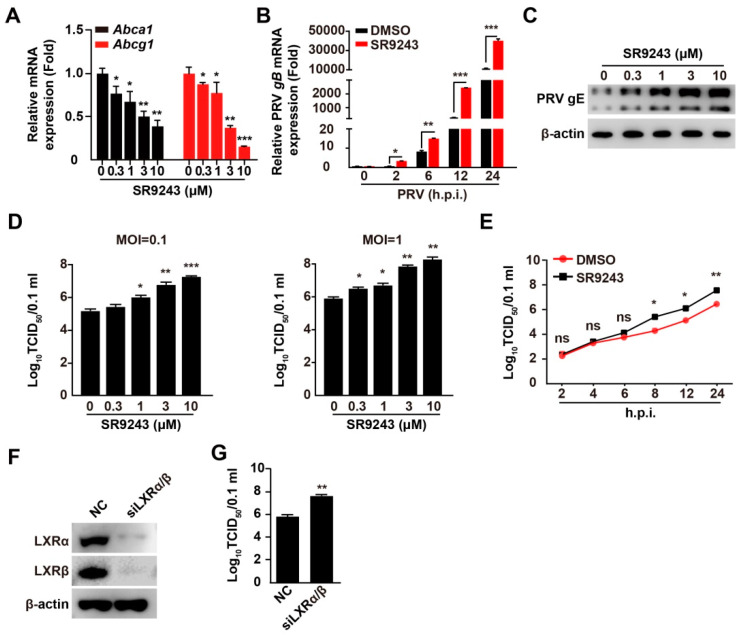
LXR inverse agonist SR9243 promotes PRV proliferation. (**A**) PK-15 cells were treated with SR9243 (0–10 µM) for 24 h. *Abca1* and *Abcg1* mRNA was assessed by qRT-PCR analysis. * *p* < 0.05, ** *p* < 0.01, *** *p* < 0.001. (**B**) PK-15 cells were infected with PRV-QXX (MOI = 0.1) and simultaneously treated with DMSO or SR9243 (10 µM) for 0–24 h. PRV *gB* mRNA was assessed by qRT-PCR analysis. * *p* < 0.05, ** *p* < 0.01, *** *p* < 0.001. (**C**) PK-15 cells were infected with PRV-QXX (MOI = 0.1) and simultaneously treated with SR9243 (0–10 µM) for 24 h. PRV gE was assessed by immunoblotting analysis. (**D**) PK-15 cells were infected with PRV-QXX (MOI = 0.1 and 1) and simultaneously treated with SR9243 (0–10 µM) for 24 h. Viral titers were assessed by a TCID_50_ assay. * *p* < 0.05, ** *p* < 0.01, *** *p* < 0.001. (**E**) PK-15 cells were infected with PRV-QXX (MOI = 0.1) and simultaneously treated with SR9243 (10 µM) for 2–24 h. One-step growth curves of PRV-QXX were assessed using a TCID_50_ assay of viral titers. * *p* < 0.05, ** *p* < 0.01. ns, no significance. (**F**) PK-15 cells were transfected with NC and siLXRα/β for 48 h. LXRα and LXRβ were assessed by immunoblotting analysis. (**G**) PK-15 cells were transfected with NC and siLXRα/β. At 24 h post-transfection, cells were infected with PRV-QXX (MOI = 1) for another 24 h. Viral titers were assessed by a TCID_50_ assay. ** *p* < 0.01.

We aimed to determine whether LXR was involved in PRV infection. SR9243 is an inverse agonist of LXR that induces LXR–co-repressor interaction and downregulates LXR-mediated gene expression [24]. As expected, treatment of PK-15 cells with SR9243 inhibited the transcription of LXR target genes, such as *Abca1* and *Abcg1* (Figure 2A). The mRNA levels of PRV *gB* in SR9243-treated cells were significantly higher than those in control cells (Figure 2B). This indicated that SR9243 promoted transcription of PRV genes. PRV gE expression was enhanced in an SR9243 dose-dependent manner (Figure 2C). We next detected the multiplication of PRV progeny virus in response to SR9243 using a viral titer assay. PK-15 cells were infected with PRV-QXX (MOI = 0.1 and 1.0) and treated with SR9243 (0–10 μM) for 24 h. SR9243 significantly promoted the production of PRV progeny virus (Figure 2D). To gain further insight into the effect of SR9243 on PRV infection, we assessed the growth kinetics of PRV under SR9243 treatment. TCID_50_ assay of viral titer indicated that SR9243 enhanced the production of the PRV progeny virus at 8 h post-treatment (Figure 2E). We also determined whether knockdown of LXRα and LXRβ improved PRV infection. Simultaneous interference LXRα and LXRβ expressions increased the production of PRV progeny virus (Figure 2F,G). These data demonstrated that inhibition of LXR benefited PRV infection.

### 3.3. Activation of LXR by Their Agonists Inhibits PRV Infection

**Figure 3:**
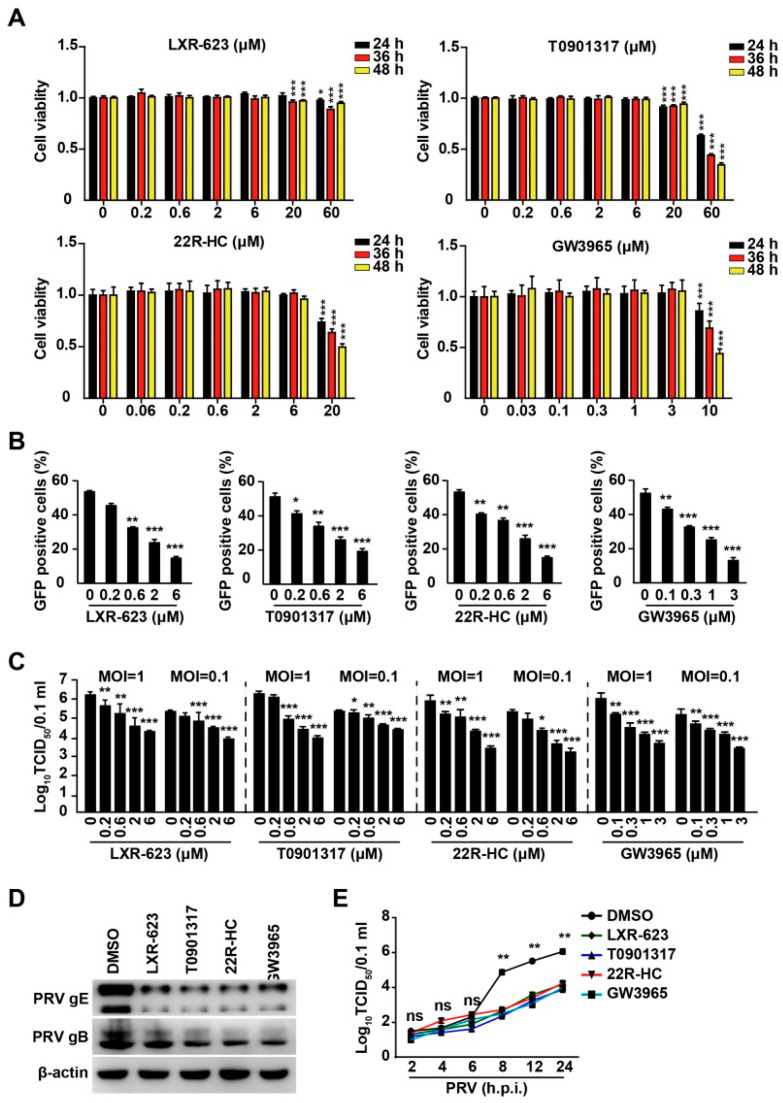
LXR agonists inhibit PRV infection. (**A**) PK-15 cells were treated with LXR-623, T0901317, 22R-HC, and GW3965 at indicated concentrations for 24–48 h. Cell viability was assessed with CCK-8 cell counting assays. * *p* < 0.05, ** *p* < 0.01, *** *p* < 0.001. (**B**) PK-15 cells were infected with PRV-GFP (MOI = 0.01) and simultaneously treated with LXR-623 (0–6 μM), T0901317 (0–6 μM), 22R-HC (0–6 μM), and GW3965 (0–3 μM) for 36 h. GFP-positive cells were analyzed by flow cytometry. * *p* < 0.05, ** *p* < 0.01, *** *p* < 0.001. (**C**) PK-15 cells were infected with PRV-QXX (MOI = 0.1 and 1) and simultaneously treated with LXR-623 (0–6 μM), T0901317 (0–6 μM), 22R-HC (0–6 μM) and GW3965 (0–3 μM) for 24 h. Viral titers were assessed by a TCID_50_ assay. * *p* < 0.05, ** *p* < 0.01, *** *p* < 0.001. (**D**) PK-15 cells were infected with PRV-QXX (MOI = 0.1) and simultaneously treated with DMSO, LXR-623 (6 μM), T0901317 (6 μM), 22R-HC (6 μM), and GW3965 (3 μM) for 24 h. PRV gB and gE were assessed by immunoblotting analysis. (**E**) PK-15 cells were infected with PRV-QXX (MOI = 0.1) and simultaneously treated with LXR-623 (6 μM), T0901317 (6 μM), 22R-HC (6 μM) and GW3965 (3 μM) for 2–24 h. One-step growth curves of PRV-QXX were assessed using a TCID_50_ assay of viral titers. ** *p* < 0.01. ns, no significance.

To confirm the negative role of LXR in PRV replication, we utilized four agonists of LXR (LXR-623, T0901317, 22R-HC, and GW3965) [25]. We first performed cell viability assays to examine the cytotoxicity of LXR agonists, and 20–60 μM of LXR-623 and T0901317 was harmful to PK-15 cells at 36–48 h post treatment (Figure 3A). 22R-HC (20 μM) and GW3965 (10 μM) showed cytotoxicity at 24–48 h post treatment (Figure 3A). LXR-623, T0901317, 22R-HC, and GW3965 resulted in decreased PRV-GFP proliferation, as indicated by flow cytometry analysis of GFP-positive cells (Figure 3B). We verified the inhibitory effect of LXR agonists on PRV infection by a viral titer assay. PK-15 cells were infected with PRV-QXX (MOI = 0.1 and 1) and treated with LXR-623 (0–6 μM), T0901317 (0–6 μM), 22R-HC (0–6 μM), and GW3965 (0–3 μM) for 24 h. Multiplication of the PRV progeny virus decreased with an increased concentration of LXR agonists (Figure 3C). PRV gB and gE expression was inhibited by LXR agonists (Figure 3D). The growth kinetics of PRV assessed by TCID_50_ assay of viral titer indicated that LXR agonists decreased the production of PRV progeny virus at 8 h post-treatment (Figure 3E). These data suggested that LXR played a negative role in PRV infection.

### 3.4. Activation of LXR Inhibits PRV Entry

**Figure 4:**
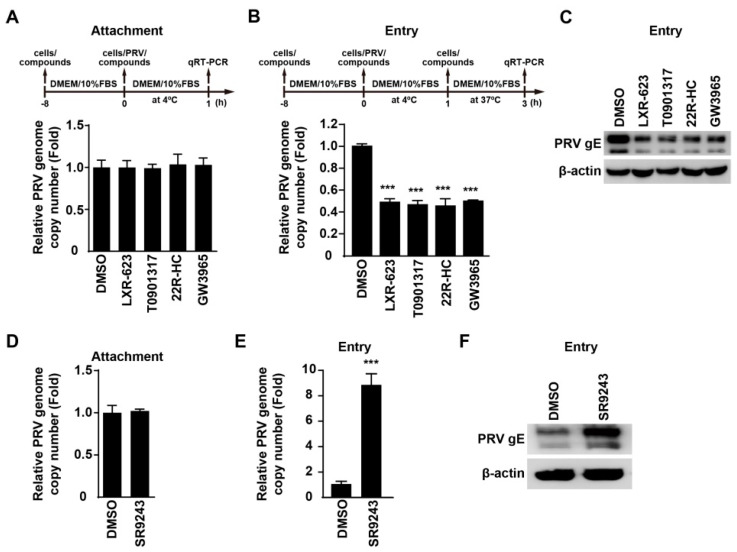
Activation of LXR inhibits PRV entry. (**A**) PK-15 cells were pretreated with DMSO, LXR-623 (6 μM), T0901317 (6 μM), 22R-HC (6 μM) and GW3965 (3 μM) for 8 h at 37 °C. Cells were incubated with PRV-QXX (MOI = 0.1) combined with DMSO, LXR-623 (6 μM), T0901317 (6 μM), 22R-HC (6 μM), and GW3965 (3 μM) for 1 h at 4 °C. After three washes with ice-cold PBS, the viral genome was isolated. PRV genome copy numbers on cells were assessed by qRT-PCR analysis. (**B**) PK-15 cells were pretreated with DMSO, LXR-623 (6 μM), T0901317 (6 μM), 22R-HC (6 μM), and GW3965 (3 μM) for 8 h at 37 °C. Cells were incubated with PRV-QXX (MOI = 0.1) combined with DMSO, LXR-623 (6 μM), T0901317 (6 μM), 22R-HC (6 μM), and GW3965 (3 μM) for 1 h at 4 °C. After three washes with ice-cold PBS, cells were cultured in prewarmed medium containing DMSO, LXR-623 (6 μM), T0901317 (6 μM), 22R-HC (6 μM), and GW3965 (3 μM) for 2 h at 37 °C. PRV genome copy numbers in cells were assessed by qRT-PCR analysis. *** *p* < 0.001. (**C**) PK-15 cells were treated as in (**B**). PRV gE in cells was assessed by immunoblotting analysis. (**D**) PK-15 cells were pretreated with DMSO and SR9243 (10 µM) for 8 h at 37 °C. Cells were then incubated with PRV-QXX (MOI = 0.1) combined with DMSO and SR9243 (10 µM) for 1 h at 4 °C. After three washes with ice-cold PBS, the viral genome was isolated. PRV genome copy numbers on cells were assessed by qRT-PCR analysis. (**E**) PK-15 cells were pretreated with DMSO and SR9243 (10 µM) for 8 h at 37 °C. Cells were incubated with PRV-QXX (MOI = 0.1) combined with DMSO and SR9243 (10 µM) for 1 h at 4 °C. After three washes with ice-cold PBS, cells were cultured in prewarmed medium containing DMSO and SR9243 (10 µM) for 2 h at 37 °C. PRV genome copy numbers in cells were assessed by qRT-PCR analysis. *** *p* < 0.001. (**F**) PK-15 cells were infected and treated as in (**E**). PRV gE in cells was assessed by immunoblotting analysis.

Next, we sought to determine which stage of viral life cycle was influenced by LXR agonists and inverse agonists in a time-of-addition assay. We examined whether activation of LXR could influence PRV attachment to cells. We pretreated PK-15 cells with LXR agonists for 8 h, and infected cells with PRV-QXX combined with LXR agonists for 1 h at 4 °C. After three washes with ice-cold PBS, we analyzed viral attachment by quantification of the PRV genome copy number by qRT-PCR analysis. LXR-623, T0901317, 22R-HC, and GW3965 did not affect PRV attachment to cells (Figure 4A). We next performed a viral entry assay by quantification of the PRV genome copy number in cells. qRT-PCR analysis indicated that LXR agonists inhibited PRV entry (Figure 4B). We also assessed PRV entry by immunoblotting analysis of PRV gE. PRV gE was decreased in cells treated with LXR agonists, which further indicated that LXR agonists inhibited PRV entry (Figure 4C).

In addition, we used LXR inverse agonists to examine whether SR9243 could promote PRV attachment and entry. qRT-PCR analysis indicated that SR9243 had no inhibitory effect on PRV attachment to cells, but it could promote PRV entry (Figure 4D,E). Immunoblotting analysis of PRV gE indicated that SR9243 increased gE in SR9243-treated cells, suggesting that SR9243 boosted PRV entry (Figure 4F). These results demonstrated that LXR was related to PRV entry.

### 3.5. PRV Infection Increases Cellular Cholesterol Content That Is Inhibited by LXR Activation

**Figure 5:**
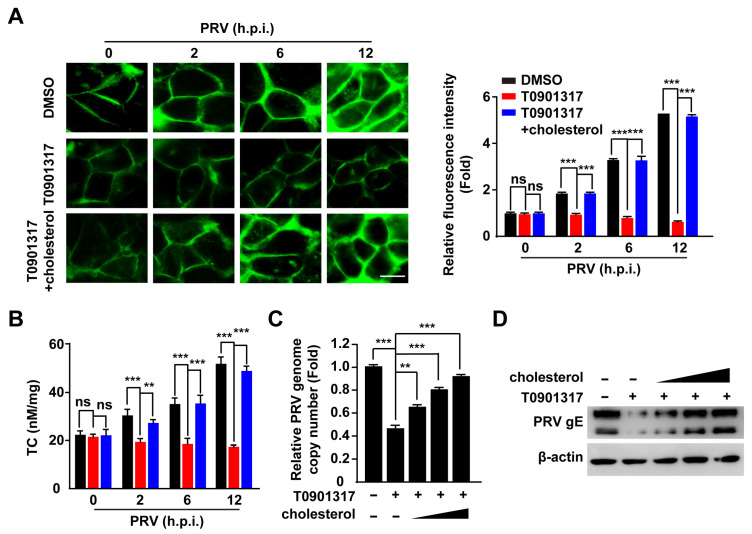
Activation of LXR decreases cellular cholesterol to inhibit PRV entry. (**A**) PK-15 cells were infected with PRV-QXX (MOI = 0.1) and simultaneously treated with DMSO, T0901317 (6 μM), and T0901317 (6 μM) + cholesterol (0.003 μg/mL) for 0–12 h. Cholesterol was detected by filipin staining (left). Quantification of the relative fluorescence intensity of filipin is shown on the right. *** *p* < 0.001. ns, no significance. Scale bar: 10 μm. (**B**) PK-15 cells were treated as in (**A**). Quantification of cellular cholesterol was performed by biochemical determination. ** *p* < 0.01, *** *p* < 0.001. ns, no significance. (**C**) PK-15 cells were incubated with PRV-QXX (MOI = 0.1) for 1 h at 4 °C and then in medium containing T0901317 (6 μM) and cholesterol (0, 0.0003, 0.001, and 0.003 μg/mL) as indicated for 2 h at 37 °C. PRV genome copy numbers in cells were assessed by qRT-PCR analysis. ** *p* < 0.01, *** *p* < 0.001. (**D**) PK-15 cells were infected and treated as in (**C**). PRV gE in cells was assessed by immunoblotting analysis.

Cholesterol is critical for PRV entry [26], so we examined cellular cholesterol content by filipin staining in PRV-infected and T0901317-treated cells. PRV infection significantly increased cellular cholesterol content (Figure 5A). However, activation of LXR by T0901317 abrogated PRV-induced enhancement of cellular cholesterol, which was restored by cholesterol replenishment (Figure 5A). This phenomenon was verified by quantification of cellular cholesterol (Figure 5B). We examined whether cholesterol replenishment rescued PRV entry during LXR activation. qRT-PCR indicated that PRV genome copy number in T0901317-treated PK-15 cells was gradually increased with the concentration of cholesterol (Figure 5C). Exogenous supplementation of cholesterol in T0901317-treated PK-15 cells restored the internalization of PRV gE, as indicated by immunoblotting analysis (Figure 5D). These data suggested that LXR influenced cellular cholesterol to inhibit PRV entry.

### 3.6. T0901317 Inhibit CCP Dynamics through Reducing Cellular Cholesterol

**Figure 6:**
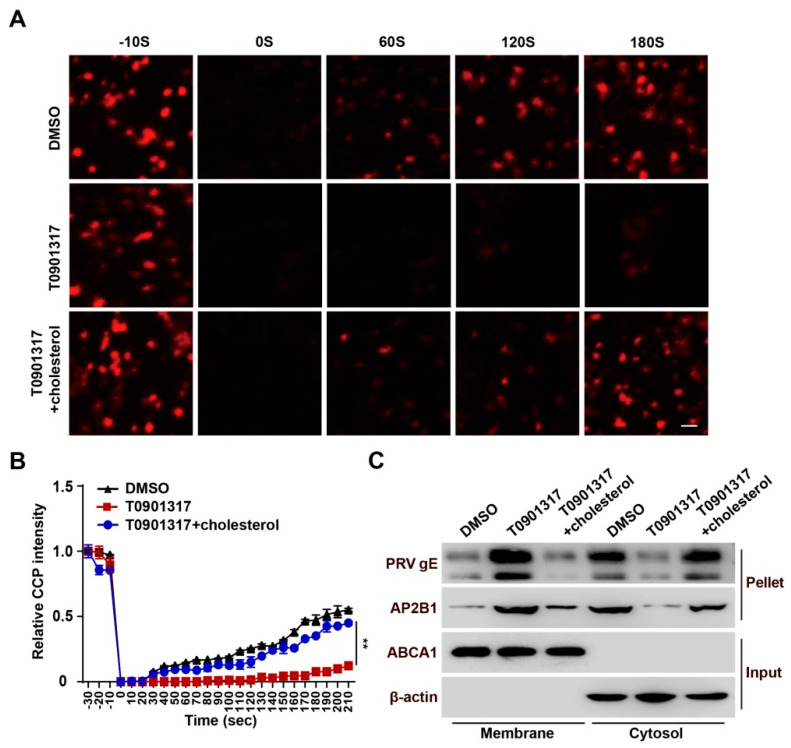
T0901317 blocks CCP dynamics-dependent viral entry. (**A**) HeLa cells were transfected with AP2B1-mCherry plasmid for 24 h followed by treatment with DMSO, T0901317 (6 μM) and T0901317 (6 μM) + cholesterol (0.003 μg/mL) for a further 8 h. The CCP dynamics were assessed by FRAP analysis. Scale bar: 1 μm. (**B**) Quantification of the relative fluorescent intensity of AP2B1 puncta in the FRAP region over time from (**A**) (*n* = 10). ** *p* < 0.01. (**C**) PK-15 cells were incubated with PRV-QXX (MOI = 0.1) for 1 h at 4 °C and then in medium containing DMSO T0901317 (6 μM) and T0901317 (6 μM) + cholesterol (0.003 μg/mL) at 37 °C. The internalization of PRV gE and AP2B1 was assessed by cell surface biotinylation assay after viral entry for 15 min.

Our previous study suggested that Niemann–Pick C1 deficiency attenuates PRV entry by decreasing cholesterol abundance and by inhibiting CCP dynamics [26]. Therefore, we examined whether LXR agonists acted via a similar mechanism. To corroborate the role of T0901317 in CCP dynamics, FRAP analysis was carried out with live-cell confocal microscopy imaging. Following the 20-s bleaching laser pulse, AP2B1-mCherry fluorescence rapidly recovered to about 50% of its initial value at approximately 180 s in control cells, while T0901317-treated cells did not show recovery of AP2B1-mCherry fluorescence (Figure 6A,B). Exogenous cholesterol was able to complement the inhibitory effect of T0901317 on the recovery of AP2B1-mCherry fluorescence (Figure 6A,B). Additionally, using a cell surface biotinylation assay, we confirmed that T0901317 had no effect on the interaction between PRV virions and AP2B1, but inhibited viral entry, which could be restored by the addition of cholesterol (Figure 6C). These data demonstrated that activation of LXR blocked PRV entry by interfering with cholesterol-dependent CCP dynamics.

### 3.7. T0901317 Prevents PRV Infection In Vivo

**Figure 7:**
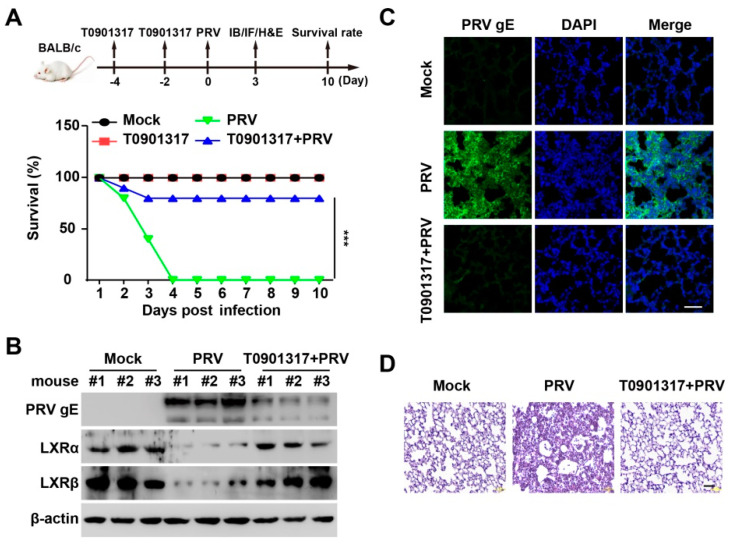
T0901317 inhibits PRV infection in vivo. (**A**) Mice were intraperitoneally injected with vehicle or T0901317 (30 mg/kg) on days 4 and 2. On day 0, the mice were mock infected or intranasally infected with PRV-QXX (5 × 10^3^ TCID_50_/50 μL per mouse). The survival rate was monitored daily for 10 days (*n* = 12 per group). *** *p* < 0.001. (**B**) PRV gE, LXRα, and LXRβ in the lungs were assessed by immunoblotting analysis at 3 days post-infection (*n* = 3 per group). (**C**) PRV gE in the lungs was assessed by immunofluorescence at 3 days post-infection (*n* = 3 per group). Scale bar: 100 μm. (**D**) The lung injury was assessed by H&E staining at 3 days post-infection (*n* = 3 per group). Scale bar: 100 μm.

To determine whether LXR agonists can be used as antivirals in vivo, we examined the protective effect of T0901317 against PRV infection. Mice were intraperitoneally injected with T0901317 twice every 2 days before the PRV challenge. Mice were intranasally infected with PRV-QXX for 10 days. We observed that all mice died at 4 days post-infection in the vehicle-treated group (Figure 7A). However, 92% of mice (10/12) survived in the T0901317-treated group (Figure 7A). Analysis of PRV gE by immunoblotting and immunohistochemistry showed that T0901317 decreased PRV gE expression in lungs, suggesting that T0901317 inhibited PRV proliferation in vivo (Figure 7B,C). Less infiltration of inflammatory cells was observed in the lungs in T0901317-treated mice than in vehicle-treated mice (Figure 7D). All the results indicated that activation of LXR prevented PRV infection in vivo.

## 4. Discussion

Viruses can manipulate lipid metabolism to facilitate their replication [27]. During infection, some viruses induce changes in cell membrane structures or utilize lipid synthetic enzymes to build a suitable microenvironment for different stages of the infection [12]. LXR controls cellular lipid homeostasis [28]. Oxysterols are endogenous ligands of LXR [29], and they promote HBV gene expression through activation of LXR [30]. Murine gammaherpesvirus 68 infection increases LXR expression, and there is not a corresponding increase in LXR target genes [17]. NDV infection activates LXR and its downstream lipogenic gene expression [13]. In this study, we found that PRV infection downregulated LXR expression both in vitro and in vivo, and LXR activation inhibited PRV infection. Our data indicated that different viruses used LXR for optimal replication through diverse mechanisms throughout their entire life cycle. Other physiological functions of LXR may be required for virus replication, as well as their roles in lipid metabolism. 

It has been demonstrated that synthetic LXR agonists restrict replication of NDV [13], HCV [15], vector-borne flaviviruses [31], chikungunya virus, and HIV [14], by altering cholesterol homeostasis. Recent evidence suggests that activation of LXR can induce cholesterol 25-hydroxylase (CH25H) mRNA and protein expression [32]. 25-hydroxycholesterol (25HC) is the enzymatic product of CH25H and exerts broad antiviral functions by inhibiting viral entry [33,34]. We have indicated that porcine CH25H acts as a host restriction factor on PRV infection by 25HC [33], so we speculated that LXR-activated CH25H expression may be responsible for inhibiting PRV infection.

Cholesterol is a critical component that determines membrane fluidity and architecture. For some viruses, cholesterol plays an essential role in the process of virus entry into cells, such as PRV [33], foot-and-mouth disease virus [35], and human rhinovirus type 2 [36], and depletion of cholesterol significantly inhibits viral entry and infection. Our data indicated that PRV enhanced cellular cholesterol levels, which were abrogated by LXR agonists. We further demonstrated that LXR activation decreased cellular cholesterol levels to inhibit PRV entry-dependent CCP dynamics, which could be rescued by cholesterol replenishment. Our study demonstrated a mechanism by which PRV disturbed LXR expression to promote viral entry through modulation of cholesterol homeostasis. This is in accordance with our previous report that cholesterol is critical for CCP dynamics in PRV entry [26]. These data provide novel insights into the prevention and control of diverse viruses that require cholesterol-regulated CCP dynamics for viral entry.

## 5. Conclusions

Viruses hijack cellular metabolism for their optimal replication. A better understanding of the interaction between virus and cellular metabolism will provide insights into an antiviral strategy. Lipids are the key component of cellular membrane compartments that participate in viral replication. We previously reported that Niemann–Pick C1-mediated intracellular cholesterol transport is essential for CCP dynamics and for viral entry. Here, we reported that PRV modulated cholesterol metabolism by downregulating the expression of LXR to assist viral entry through clathrin-mediated endocytosis. Therefore, our data further validated that cholesterol-regulated CCP dynamics are pivotal for PRV infection, suggesting that pharmacological reduction of cellular cholesterol levels has the potential to prevent PRV infection.

## Data Availability

All available data are presented in the article.
